# A Propensity Score Analysis of Chemotherapy Use in Patients With Resectable Gallbladder Cancer

**DOI:** 10.1001/jamanetworkopen.2021.46912

**Published:** 2022-02-16

**Authors:** Muhammet Ozer, Suleyman Y. Goksu, Nina N. Sanford, Matthew Porembka, Hajra Khurshid, Chul Ahn, Mary Claire Maxwell, Muhammad Shaalan Beg, Syed M. Kazmi

**Affiliations:** 1 Division of Hematology and Oncology, University of Texas Southwestern Medical Center, Dallas; 2 Department of Internal Medicine, Capital Health Regional Medical Center, Trenton, New Jersey; 3 Department of Radiation Oncology, University of Texas Southwestern Medical Center, Dallas; 4 Division of Surgical Oncology, Department of Surgery, University of Texas Southwestern Medical Center, Dallas; 5 Department of Internal Medicine, University of Texas Southwestern Medical Center, Dallas; 6 Department of Population and Data Sciences, University of Texas Southwestern Medical Center, Dallas

## Abstract

**Importance:**

Gallbladder cancer is uncommon but highly fatal. Surgery remains the only potentially curative treatment for localized or locoregionally advanced gallbladder cancer. The rate of use of neoadjuvant and adjuvant chemotherapy in resectable gallbladder cancer is unknown.

**Objective:**

To assess factors associated with the use of neoadjuvant and adjuvant chemotherapy in patients with resectable gallbladder cancer and survival outcomes.

**Design, Setting, and Participants:**

The National Cancer Database was used to identify 6391 adults who underwent definitive surgical resection for gallbladder cancers between January 1, 2004, and January 1, 2016. Data analysis was performed from January 15 to February 15, 2020. Patients with localized or locoregionally advanced gallbladder cancers (ie, categories cTx-cT4, cN0-2, and cM0) were categorized as receiving neoadjuvant chemotherapy, adjuvant chemotherapy, or surgery alone. Categorical variables were compared using the χ^2^ test, with 1:3 nearest-neighbor propensity score matching based on neoadjuvant chemotherapy. Survival outcomes between groups were compared using Kaplan-Meier and Cox proportional hazards regression analyses.

**Main Outcomes and Measures:**

The use and survival outcomes of adjuvant and neoadjuvant chemotherapy.

**Results:**

Of 6391 patients who underwent definitive surgery for gallbladder cancer, 4559 were women (71.3%); median age was 68 (IQR, 59-77) years. A total of 3145 patients (49.2%) received adjuvant chemotherapy, 3145 patients (49.2%) underwent surgery without chemotherapy, and 101 patients (1.6%) received neoadjuvant chemotherapy. Neoadjuvant chemotherapy use was associated with treatment at an academic facility (61 patients [60%] vs 38 patients [38%] treated in a nonacademic facility; *P* < .001) and in those with private insurance (65 patients [65%] vs 11 patients [11%] with Medicaid insurance; *P* < .001). Surgery alone was frequently used in older patients (median age, 72 [IQR, 63-81] years vs 59 [IQR, 52-66] years in patients with neoadjuvant chemotherapy; *P* < .001), those with Medicare insurance (1925 patients [57%] vs 1438 patients [43%] with adjuvant chemotherapy; *P* < .001), and patients with a higher comorbidity index score (326 patients [62%] vs 197 patients [38%] with adjuvant chemotherapy; *P* < .001). Adjuvant or neoadjuvant chemotherapy was used more frequently than surgery in patients with node-positive cancer (1482 [67.2%] vs 53 [65.4%] vs 912 [49.7%]). On propensity score matching analysis, adjuvant chemotherapy was associated with longer survival than surgery alone (22 vs 18 months, hazard ratio [HR], 0.78; 95% CI, 0.63-0.96); survival with neoadjuvant chemotherapy was not statistically significant compared with surgery alone and adjuvant chemotherapy groups (27 months, HR, 0.78; 95% CI, 0.58-1.04). However, in patients with node-positive gallbladder cancer, neoadjuvant therapy was associated with longer median overall survival (30 months [95% CI, 24-36 months] vs 14 months [95% CI, 11-17] in patients with surgery alone; *P* = .002).

**Conclusions and Relevance:**

In this cohort study, use of adjuvant and neoadjuvant chemotherapy was low in patients with surgically resected gallbladder cancers. Chemotherapy was used more frequently than surgery in lymph node–positive disease compared with lymph node–negative disease. Adjuvant chemotherapy was associated with a survival advantage in resectable gallbladder cancer, and neoadjuvant chemotherapy was associated with increased survival in node-positive gallbladder cancers. These findings suggest that adjuvant chemotherapy and neoadjuvant chemotherapy should be considered in treatment of gallbladder cancer.

## Key Points

**Question:**

What are the characteristics and survival outcomes associated with neoadjuvant and adjuvant chemotherapy in patients with resectable gallbladder cancer?

**Findings:**

In this cohort study including 6391 patients, 49.2% of the patients received adjuvant chemotherapy and 1.6% received neoadjuvant chemotherapy. Adjuvant chemotherapy was associated with statistically significant improvement in survival in patients with gallbladder cancer; neoadjuvant chemotherapy was associated with the greatest benefit in node-positive disease.

**Meaning:**

The findings of this study suggest that adjuvant chemotherapy and neoadjuvant chemotherapy should be considered in patients with resectable gallbladder cancer.

## Teaser

This cohort study examines the use and outcomes associated with neoadjuvant and adjuvant chemotherapy vs surgery alone in patients with resectable gallbladder cancer.

## Introduction

Gallbladder cancer is a highly aggressive disease with a late presentation and poor prognosis.^1,2^ Complete surgical excision remains the only potentially curative treatment for early-stage gallbladder cancer.^3,4^ Adjuvant chemotherapy is used in some patients, with gemcitabine plus cisplatin being the most commonly used adjuvant regimen; however, to our knowledge, no phase 3 randomized clinical trials have evaluated this regimen.^5,6^ In intrahepatic, hilar cholangiocarcinoma, or muscle-invasive gallbladder cancers, the Capecitabine or Observation After Surgery in Treating Patients With Biliary Tract Cancer (BILCAP) trial of adjuvant capecitabine starting within 12 weeks postoperatively and given twice a day on days 1 to 14 of a 3-weekly cycle for 24 weeks (8 cycles) vs observation failed to reach its intention-to-treat primary end point of improvement in median overall survival.^7^ One limitation of the BILCAP trial is that the adherence to adjuvant chemotherapy was 55%. In other aggressive tumors with the similar dilemma of early recurrence and poor tolerance to adjuvant chemotherapy, neoadjuvant chemotherapy has provided several advantages.^8,9,10,11,12^ These advantages include early management of micrometastatic disease, identifying patients with chemotherapy-responsive disease, downstaging tumors leading to margin-negative resection, and avoiding surgical resection in patients with early progression.^3^ Neoadjuvant chemotherapy is now a preferred approach in several gastrointestinal cancers, such as pancreatic, gastric, and rectal cancer. The use and benefit of chemotherapy in resectable gallbladder cancer, especially in the neoadjuvant setting, is not well studied in the US and, to our knowledge, neoadjuvant chemotherapy has not been evaluated in any randomized clinical trial. In this study, we report on the use and outcomes with chemotherapy, both in the neoadjuvant and adjuvant settings, compared with surgery alone in patients with localized or locoregionally advanced but resectable gallbladder cancers.

## Methods

We conducted this retrospective cohort study by querying the National Cancer Database (NCDB), which is one of the largest cancer databases in the US and includes approximately 70% of cancer cases. The NCDB contains deidentified patient data with sociodemographic status, tumor characteristics, and treatment variables.^13^ This study was exempted from institutional review board approval given the use of publicly available deidentified data according to the US Department of Health and Human Services (45 CFR §46). This study followed the Strengthening the Reporting of Observational Studies in Epidemiology (STROBE) reporting guideline.

**Figure 1.**
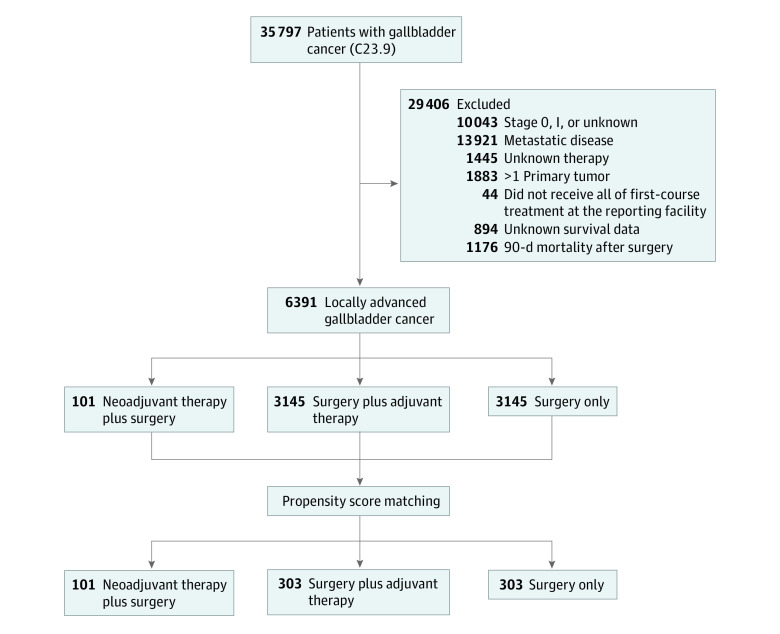
Criteria for Selection of Patients With Locally Advanced Gallbladder Cancer C23.9 indicates *International Classification of Diseases for Oncology, Third Edition*/World Health Organization 2008 site recode for patients with gallbladder cancer.^16^

We identified adult patients (age ≥18 years) with localized or locoregionally advanced gallbladder cancers who underwent definitive surgery between January 1, 2004, and January 1, 2016.^14,15^ We used the C23.9 *International Classification of Diseases for Oncology, Third Edition*/World Health Organization 2008 site recode for patients with gallbladder cancer.^16^ We identified patients who underwent definitive surgery using the Facility Oncology Registry Data Standards codes 30 to 80. We included patients with stage II to III and stage IV (with no distant metastasis: cTx-cT4, cN0-2, and cM0). We excluded those who did not undergo definitive surgery (no surgery, local tumor destruction, not specified, or unknown), had metastatic disease (M1), did not receive all first-course treatment at the reporting facility, had unknown survival data or treatment, and had more than 1 primary tumor (Figure 1). In addition, to minimize immortal time bias, we excluded patients who died within 90 days from the time of the definitive surgery. Three groups were identified based on chemotherapy use: surgery alone, adjuvant chemotherapy, and neoadjuvant chemotherapy. Other variables included were age at diagnosis, sex (male, female), race and ethnicity (Hispanic, non-Hispanic Black, and non-Hispanic White), Charlson Comorbidity Index score (0 to ≥2), facility type (academic, nonacademic), median income quartiles, insurance status (uninsured, private insurance, Medicaid, Medicare), and NCDB analytic stage group (II-IV). The NCDB analytical stage group is defined as a pathological stage group; however, the clinical stage group was used if the pathological group is absent. Data on race and ethnicity were not required by the funding agency; however, we provided the data because they are important as demographic information for patients with cancer.

### Statistical Analysis

Data analysis was conducted from January 15 to February 15, 2020. Baseline characteristics of treatment groups were compared using the χ^2^, Fisher exact, and analysis of variance tests. Propensity scores were estimated by using logistic regression models. A 2-group propensity score matching was performed sequentially using a 1:3 nearest-neighbor matching algorithm to minimize selection bias after adjusting for age at diagnosis, sex, race, ethnicity, Charlson Comorbidity Index score, and stage.^17,18^ First, surgery alone and neoadjuvant chemotherapy groups were matched, and then adjuvant chemotherapy and neoadjuvant chemotherapy groups were matched.^17^ Because only a small number of patients received neoadjuvant chemotherapy, we selected 1 to multiple (1:3) matching to include more patients in the analysis. Survival outcomes are reported after propensity score matching. We used propensity score matching over inverse probability weighted as we aimed to estimate the average treatment effect for patients. We present age as median (IQR) and survival as median (95% CI) values. Overall survival was calculated using the adjusted Kaplan-Meier method with inverse probability of treatment weighted to reduce confounding effects. We present the overall survival plot using the inverse probability-weighted Kaplan-Meier method.^19^ A multivariable Cox proportional hazards regression model was performed to assess treatment variables as independent factors associated with survival outcomes. Missing data were coded as unknown variables and included in the multivariable Cox proportional hazards regression analysis. SPSS, version 22 (SPSS Inc) and R software, version 3.6.2 with the MatchIt package (R Foundation for Statistical Computing) were used for the propensity score–matched analyses.^20,21,22^ Covariate balance was calculated using standardized differences after propensity score matching, and covariables were considered well balanced if the standardized differences were less than 0.2.^23^ The covariates were well balanced among the groups. Findings were considered statistically significant at *P* < .05.

## Results

A total of 6391 patients were identified who matched our criteria. Of these, 1832 were men (28.7%) and 4559 were women (71.3%); median age was 68 (IQR, 59-77) years. Surgery alone was used in 3145 patients (49.2%), and an equal number of patients (3145 [49.2%]) received adjuvant chemotherapy. Only a small proportion of patients (101 [1.6%]) received neoadjuvant chemotherapy (Figure 1). Use of neoadjuvant therapy was more frequent in younger patients (median age, 59 [IQR, 52-66] years) compared with surgery alone (median age, 72 [IQR, 63-81] years) or adjuvant chemotherapy (median age, 65 [IQR, 57-72] years) (*P* < .001). Use of neoadjuvant chemotherapy (77 [76.2%]) and adjuvant chemotherapy (2284 [72.6%]) was higher in patients with a Charlson Comorbidity Index score of 0, compared with surgery alone (2033 [64.6%]) (*P* < .001). Surgery alone was frequently used in patients with a higher comorbidity index score (326 patients [62%] vs 197 patients [38%] with adjuvant chemotherapy; *P* < .001). The use of adjuvant chemotherapy was frequent in patients with node-positive cancer (1482 [67.2%]). Use of neoadjuvant chemotherapy was frequent in patients with private insurance rather than other insurance groups (65 patients [65%] vs 11 patients [11%] Medicaid insurance; *P* < .001), reflecting its use in younger patients, and adjuvant chemotherapy (1438 patients [43%]) and surgery alone (1925 patients [57%]) were frequent in patients with Medicare insurance. Neoadjuvant chemotherapy was more likely to be given in academic centers (61 patients [60%]) compared with nonacademic settings (38 patients [38%]) (*P* < .001).

**Figure 2.**
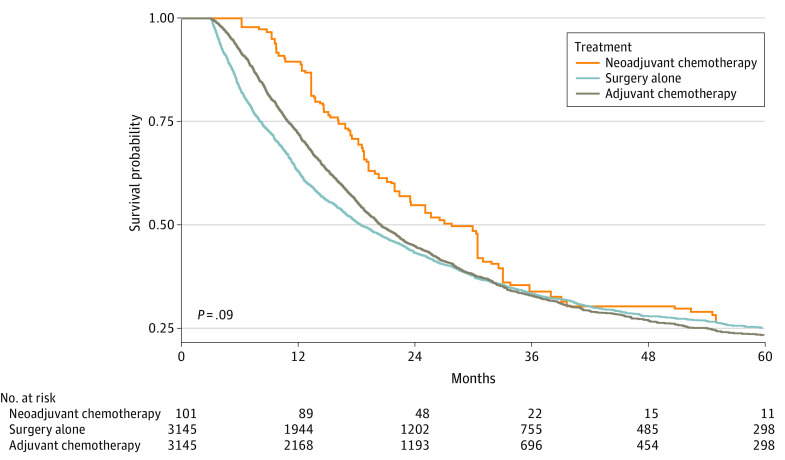
Overall Survival by Treatment Groups in Using Inverse Probability Weighted Kaplan-Meier Method

A total of 101 patients who received neoadjuvant chemotherapy were matched with 303 patients in each adjuvant chemotherapy and surgery-alone group. After propensity score matching, most differences between the groups became nonsignificant except receiving treatment in academic vs nonacademic facilities. For neoadjuvant chemotherapy 61 [19.7%] vs 38 [10.2%], for surgery alone 123 [39.8%] vs 168 [45.0%], and for adjuvant chemotherapy 125 [40.5%] vs 167 [44.8%]) received treatment in academic vs nonacademic facilities (*P* = .002) (Table 1 and Table 2). Multivariable analysis demonstrated that, compared with surgery alone, adjuvant chemotherapy was associated with a statistically significant improvement in overall survival (22 vs 18 months: hazard ratio [HR], 0.78; 95% CI, 0.63-0.96) (Table 3); overall survival is shown in Figure 2. Despite higher absolute median survival in the neoadjuvant chemotherapy group, this difference was not statistically significant, possibly owing to a smaller sample size (27 vs 18 months: HR, 0.78; 95% CI, 0.58-1.04). Other factors associated with decreased survival were a higher stage (stage IV: HR, 2.04; 95% CI, 1.53-2.70) and the presence of positive lymph nodes (HR, 1.84; 95% CI, 1.43-2.36) (Table 3). In node-positive gallbladder cancer, longer median overall survival was associated with neoadjuvant chemotherapy (median survival time, 30 months [95% CI, 24-36 months]) compared with adjuvant chemotherapy (median survival time, 22 months [95% CI, 19-26 months]) and surgery alone (median survival time, 14 months [95% CI, 11-17]) (*P* = .002). In patients with category T3/T4 disease, neoadjuvant chemotherapy was not associated with a statistically significant difference in median overall survival (median survival time, 20 months [95% CI, 14-26 months]) compared with surgery alone (median survival time, 13 months [95% CI, 10-16 months]) and adjuvant chemotherapy (median survival time, 18 months [95% CI, 15-21 months]) (*P* = .19).

**Table 1.** Baseline Characteristics Among Treatment Groups Before Propensity Score Matching

Characteristics	No. (%)			*P* value
	Neoadjuvant	Surgery alone	Adjuvant	
No. (%)	101 (1.6)	3145 (49.2)	3145 (49.2)	
Age, median (IQR), y	59 (52-66)	72 (63-81)	65 (57-72)	<.001
Sex				
Male	32 (1.7)	900 (49.1)	900 (49.1)	.80
Female	69 (1.5)	2245 (49.2)	2245 (49.2)	
Race and ethnicity^a^				
Hispanic	>12 (>1.6)	406 (53.3)	343 (45.0)	.06
Non-Hispanic				
Black	<11 (<1.3)	392 (46.4)	442 (52.4)	
White	66 (1.6)	2024 (49.4)	2004 (48.9)	
Insurance status				<.001
Uninsured	<11 (<4.8)	112 (48.9)	116 (50.7)	
Private	65 (3.1)	790 (38.1)	1220 (58.8)	
Medicaid	11 (2.0)	247 (45.6)	284 (52.4)	
Medicare	>13 (>0.1)	1925 (56.9)	1438 (42.5)	
Income, $				
<40 227	18 (1.4)	675 (53.2)	576 (45.4)	.02
40 227-50 353	15 (1.1)	696 (49.7)	688 (49.2)	
50 354-63 332	26 (1.7)	727 (47.6)	774 (50.7)	
≥63 333	40 (1.9)	1001 (47.6)	1060 (50.5)	
Charlson Comorbidity Index score				
0	77 (1.8)	2033 (46.3)	2284 (52.0)	<.001
1	>13 (>1.0)	786 (53.4)	664 (45.1)	
≥2	<11 (<2.1)	326 (62.0)	197 (37.5)	
Facility type				
Academic	61 (2.4)	1181 (46.3)	1310 (51.3)	<.001
Nonacademic	38 (1.0)	1924 (51.6)	1767 (47.4)	
Nodal status				
Negative	28 (1.7)	925 (55.2)	722 (43.1)	<.001
Positive	53 (2.2)	912 (37.3)	1482 (60.6)	
No nodes examined	18 (0.8)	1294 (57.8)	928 (41.4)	
Margin				
R0	75 (1.9)	2088 (52.2)	1836 (45.9)	.13
R1	<11 (<1.1)	418 (43.5)	532 (55.4)	
R2	<11 (<7.4)	61 (40.9)	87 (58.4)	
Stage				
II	31 (1.0)	1850 (61.8)	1111 (37.1)	<.001
III	51 (2.2)	939 (41.0)	1298 (56.7)	
IV	19 (1.7)	356 (32.0)	736 (66.2)	
Overall survival, median (95% CI)	27.0 (20.5-33.6)	17.6 (12.4-22.8)	22.3 ()19.4-25.3	.13

^a^ Race and ethnicity data were self-reported; for this study, terms were classified for reporting convenience.

**Table 2.** Baseline Characteristics Among Treatment Groups After Propensity Score Matching^a^

Characteristics	No. (%)			SMD^b^	
	Neoadjuvant	Surgery alone	Adjuvant	Neoadjuvant vs adjuvant	Neoadjuvant vs surgery alone
No. (%)	101 (14.3)	303 (42.9)	303 (42.9)	NA	NA
Age, median (IQR), y	59 (36-87)	59 (21-87)	60 (31-83)	−0.07	−0.02
Sex					
Male	32 (14.5)	99 (44.8)	90 (40.7)	−0.02	0.01
Female	69 (14.2)	204 (42.0)	213 (43.8)		
Race and ethnicity					
Hispanic	>12 (>16.7)	28 (38.9)	31 (43.1)	0.02	0.04
Non-Hispanic					
Black	<11 (<16.2)	>30 (>44.2)	27 (39.7)	0.01	0.003
White	66 (13.0)	211 (41.5)	232 (45.6)	−0.1	−0.04
Insurance status					
Uninsured	<11 (<26.8)	>11 (>26.8)	19 (46.3)	NA	NA
Private	65 (17.5)	152 (40.9)	155 (41.7)	NA	NA
Medicaid	11 (15.3)	35 (48.6)	26 (36.1)	NA	NA
Medicare	>13 (>6.4)	89 (43.8)	91 (44.8)	NA	NA
Income, $					
<40 227	18 (14.2)	58 (45.7)	51 (40.2)	NA	NA
40 227-50 353	15 (9.4)	78 (48.8)	67 (41.9)	NA	NA
50 354-63 332	26 (14.4)	63 (34.8)	92 (50.8)	NA	NA
≥63 333	40 (17.9)	97 (43.3)	87 (38.8)	NA	NA
Charlson Comorbidity Index score					
0	77 (13.6)	250 (44.2)	238 (42.1)	−0.02	−0.06
1	24 (16.2)	53 (39.2)	65 (44.6)	0.02	0.04
≥2	3 (25.0)	2 (16.7)	7 (58.3)	0.007	0.02
Facility type					
Academic	61 (19.7)	123 (39.8)	125 (40.5)	NA	NA
Nonacademic	38 (10.2)	168 (45.0)	167 (44.8)	NA	NA
Nodal status					
Negative	28 (14.2)	86 (43.7)	83 (42.1)	NA	NA
Positive	53 (16.7)	119 (37.4)	146 (45.9)	NA	NA
No nodes examined	18 (9.5)	97 (51.3)	74 (39.2)	NA	NA
Margin					
R0	75 (17.2)	178 (40.8)	183 (42.0)	NA	NA
R1/R2	11 (9.4)	50 (42.7)	56 (47.9)	NA	NA
Stage					
II	31 (13.8)	104 (46.2)	90 (40.0)	0.01	−0.04
III	51 (14.0)	150 (41.1)	164 (44.9)	−0.04	0.01
IV	19 (16.2)	49 (41.9)	49 (41.9)	0.03	0.03

Abbreviations: NA, not applicable; SMD, standardized mean difference.

^a^ Adjusted for age at diagnosis, sex, race, ethnicity, Charlson Comorbidity Index score, and cancer stage.

^b^ Considered balanced if the value was less than 0.2.

**Table 3.** Multivariable Cox Proportional Hazards Regression Analysis Among Treatment Groups in the Patients With Gallbladder Cancer After Propensity Score Matching

Characteristics	Multivariable analysis for overall survival, HR (95% CI)
Therapies	
Surgery alone	1 [Reference]
Neoadjuvant	0.78 (0.58-1.04)
Adjuvant	0.78 (0.63-0.96)
Age	1.00 (0.99-1.02)
Sex	
Male	1 [Reference]
Female	0.92 (0.75-1.12)
Race and ethnicity	
Hispanic	0.83 (0.58-1.19)
Non-Hispanic	
White	1 [Reference]
Black	0.80 (0.56-1.13)
Charlson Comorbidity Index score	
0	1 [Reference]
1	1.21 (0.95-1.53)
≥2	1.62 (0.86-3.02)
Facility type	
Academic	1 [Reference]
Nonacademic	1.18 (0.97-1.43)
Insurance status	
Uninsured	1 [Reference]
Private	1.38 (0.83-2.31)
Medicaid	1.65 (0.94-2.92)
Medicare	1.63 (0.95-2.81)
Income, $	
<40 227	1 [Reference]
$0 227-50 353	1.13 (0.84-1.51)
50 354-63 332	1.06 (0.79-1.42)
≥63 333	1.00 (0.75-1.33)
Stage	
II	1 [Reference]
III	1.46 (1.16-1.83)
IV	2.04 (1.53-2.70)
Nodal status	
Negative	1 [Reference]
Positive	1.84 (1.43-2.36)

## Discussion

The main finding of this study is that the use of chemotherapy in resectable gallbladder cancer remains below 50%. When chemotherapy is given, the adjuvant approach is the main strategy in the US.^24^ In this study, adjuvant chemotherapy was associated with improved survival in resectable gallbladder cancers compared with surgery alone. The use of neoadjuvant chemotherapy is infrequent in gallbladder cancers in the US.^25^ We also explored the factors associated with the use of neoadjuvant and adjuvant chemotherapy in the population and found that insurance status, age, and treatment facility settings were associated with their use. This study fills the gap in information about the use of neoadjuvant and adjuvant chemotherapy in real-world settings.

Although previous studies did not demonstrate a statistically significant benefit of adjuvant chemotherapy in this disease,^26,27^ the BILCAP trial showed a clinically meaningful benefit.^7^ The BILCAP trial was a phase 3 randomized clinical trial of adjuvant capecitabine treatment in the treatment of biliary tract cancers, and 18% of the patients had gallbladder cancer. The overall survival in that study was 51.1 (95% CI, 34.6-59.1) months in the capecitabine arm, which was not statistically significant, but it was a substantial absolute difference compared with 36.4 (95% CI, 29.7-44.5) months in the observation arm. The BILCAP trial was practice-changing even though it failed to reach its primary end point of improvement in median overall survival in the intention-to-treat analysis (HR, 0.81; 95% CI, 0.63-1.04). The overall survival in our current real-world database analysis is lower compared with the clinical trial results, likely because clinical trials select patients based on strict inclusion criteria.

Neoadjuvant chemotherapy presents a potential strategy to counter the lower use of adjuvant chemotherapy and has several advantages.^25,28,29^ In the present analysis, the median overall survival was numerically higher in the neoadjuvant therapy group than the adjuvant therapy or surgery alone groups, but the survival outcome did not reach statistical significance, possibly because of the small sample size of patients receiving neoadjuvant therapy. Clinical benefit with neoadjuvant chemotherapy was noted in patients with node-positive gallbladder cancer, with survival significantly higher in the neoadjuvant therapy group (30 months) vs the adjuvant chemotherapy (22 months) and surgery alone (14 months) groups. Therefore, neoadjuvant chemotherapy in resectable gallbladder cancers may be considered an important area to study in patients with possible lymph node involvement. Lymph node regression is a noted factor associated with the histopathological and radiological response to neoadjuvant chemotherapy in gallbladder cancer. Agrawal et al^30^reported overall radiological response rates in patients with lymph node involvement of 72.6% in those with N1 disease (lymph nodes close to cystic duct, common bile duct, hepatic artery, or portal vein), 75% in patients with N2 disease (aortic, caval, superior mesenteric artery, or celiac nodes), and 59.9% in patients with para-aortic lymphadenopathy who received neoadjuvant chemotherapy. Other disease-related factors that may benefit from the neoadjuvant approach include T3/T4 disease, such as involvement of the hepatic artery, portal vein, or adjacent organs without distant metastasis.^14,15^ The present study also showed higher (but not statistically significant) overall survival in patients with T3/T4 disease treated with neoadjuvant chemotherapy.

Neoadjuvant chemotherapy in gallbladder cancer potentially allows selection of more favorable disease characteristics, achieving a higher R0 rate and early control of systemic disease by targeting micrometastasis and potentially sparing surgery in patients who experience early recurrence.^25^ There is no consensus definition of patients at such high risk, but other groups have reported greater than or equal to T3 tumor categories, poorly differentiated, and node-positive disease as acceptable criteria for neoadjuvant chemotherapy^31^ that can allow achievement of an R0 resection.^32^ de Aretxabala et al^33^ performed a phase 2 study including 18 patients with locally advanced or borderline resectable gallbladder cancer who received neoadjuvant fluorouracil plus radiotherapy and reported 86% resectability with 24 months’ survival of 46%. Similarly, Creasy et al^34^ reported that 45% of patients with locally advanced gallbladder cancer were able to undergo R0 resection post neoadjuvant therapy, with a median overall survival of 51 months in the R0 group vs 11 months in the other groups. Another retrospective study of neoadjuvant chemotherapy in patients with locoregionally advanced gallbladder cancer reported a 41.2% resection rate.^14^ Patients who underwent curative resection had a median overall survival of 49 months vs 13 months for the whole cohort. Other positive results with neoadjuvant chemotherapy have been demonstrated in a few other cohorts.^35,36,37^ A phase 3 randomized clinical trial (NCT02867865) of perioperative therapy in patients with locally advanced gallbladder cancer designed to evaluate the survival effect of neoadjuvant chemotherapy alone vs chemoradiotherapy is recruiting.^15^

There is a need for improvement of clinical outcomes in gallbladder cancers. Future clinical trials can focus on exploring the neoadjuvant chemotherapy approach at least in node-positive disease identified at baseline imaging. It may be also important to separate the biliary cancers from gallbladder cancers in light of molecular differences seen in these cancers. Gallbladder cancers had higher rates of homologous recombination repair deficiency and *ERBB2* (formerly *HER2/neu*) overexpression and amplification.^38^ Targeted therapies may be incorporated earlier in clinical care that may improve results in this cancer. The role of radiotherapy in these cancers remains unclear and can be a future area of research.

### Limitations

This study had limitations, including the retrospective design. Excluding patients who had unknown survival status may have introduced selection bias into the analysis; however, survival analysis is necessary, and unknown follow-up data may lead to bias. Propensity score matching adjusts for confounding by the matched variables; however, the study is still vulnerable to unmeasured confounding. In addition, the number of patients in the neoadjuvant group was small. The NCDB provides data on the first course of treatment and lacks details on chemotherapy regimens, the number of cycles, and doses. Also, data on toxic and adverse events for chemotherapy regimens are not included in the NCDB. The Charlson Comorbidity Index score is available in the NCDB; however, there is no information on performance status.

## Conclusions

The use of both adjuvant and neoadjuvant chemotherapy was low in this study. Adjuvant chemotherapy was associated with statistically significant improvement in survival in patients with gallbladder cancer. Neoadjuvant chemotherapy was not significantly associated with improved median overall survival compared with adjuvant therapy or surgery alone, but patients with node-positive disease experienced significantly longer median survival with neoadjuvant treatment. These findings suggest that use of adjuvant chemotherapy and neoadjuvant chemotherapy should be considered in the treatment of gallbladder cancer.
